# Does the clinical and radiologic outcomes following total knee arthroplasty using a new design cobalt-chrome tibial plate or predecessor different?

**DOI:** 10.1186/s43019-024-00239-0

**Published:** 2024-11-12

**Authors:** Kang-Il Kim, Jun-Ho Kim, Kyeonguk Min

**Affiliations:** 1 Department of Orthopaedic Surgery, Center for Joint Diseases, Kyung Hee University Hospital at Gangdong, Seoul, Korea; 2 Department of Orthopaedic Surgery, School of Medicine, Kyung Hee University, Seoul, Korea

**Keywords:** Knee arthroplasty, Radiolucent line, Tibial base plate, Cementation technique

## Abstract

**Background:**

This study aimed to compare clinical and radiographic outcomes for a new tibial component (Attune S +) and the previous design (Attune S) in total knee arthroplasty (TKA) patients using ATTUNE® posterior stabilized (PS) prosthesis and also assessed related factors for the development of tibial radiolucent line(RLL).

**Methods:**

This retrospective study included 362 knees (179 Attune S, 183 Attune S +) with an average 4 years (range, 2–8) follow-up. Clinical outcomes, radiologic parameters and the incidence of RLL around the tibial component were compared through the serial assessment. For the subgroup analysis, radiologic parameters were compared between patients with and without RLL.

**Results:**

There was no significant difference in terms of clinical outcomes and radiologic parameters between two designs. The incidence of RLL was not different through the serial follow-up (*P* > 0.05). In the subgroup analysis, the preoperative medial proximal tibial angle (83.7° versus 85.0°, *P* = 0.01) was smaller and preoperative hip-knee-ankle angle (169.1° versus 171.8°, *P* = 0.01) has more varus in the group with RLL than those without.

**Conclusions:**

The clinical and radiologic outcomes including the incidence of tibial RLL between new design and predecessor were not significantly different at average 4 years follow-up. The development of tibial RLL was associated with preoperative varus deformity of tibia and lower limb alignment.

**Supplementary Information:**

The online version contains supplementary material available at 10.1186/s43019-024-00239-0.

## Introduction

Since John Insall and his colleagues first conceived the prototype of total knee arthroplasty in 1973, there have been various design changes in the artificial joint devices to achieve better surgical outcomes. However, implant loosening and long-term survival remain significant issues [1]. Recently, early failure of the tibial component has been reported in patients who received TKA using the initial design of ATTUNE^®^ posterior stabilized (PS) prosthesis (Depuy Synthes, Warsaw, IN, USA) [2, 3]. RLLs often indicate the onset of bone weakening and loss of adhesion to cement, which can lead to the implant loosening [4]. Furthermore, the design of ATTUNE prosthesis showed higher incidence of radiolucent line (RLL) on postoperative radiographs compared with its predecessor (PFC, DePuy Synthes, Warsaw, IN, USA), which is closely related to stress shielding. This might be caused that the implant made of cobalt chrome (CoCr) alloy is more rigid than titanium alloy, and the tibial baseplate is thicker than that of other implants [5].

To address this concern, a new design of tibial plate (Attune S +) has been introduced, which has more cement pockets, increased surface roughness value, and reduced thickness of tibial baseplate [6]. In the previous of in vitro studies for the Attune S + , it was reported that the surface roughness of the tibial plate increased due to changes in the surface treatment, leading to an increase in pull-out strength [7]. Moreover, a recent comparative study reported that the new tibial baseplate was more advantageous in preventing medial proximal tibial resorption than the original baseplate [5]. However, there is still lack of evidence whether the clinical and radiologic outcomes including aseptic loosening of tibial component was significantly different between the two tibial designs with a mid-term follow-up.

Therefore, current study aims to compare the clinical and radiologic outcomes with a serial radiographic evaluation and its survival in patients with TKA using between the first and the second generation of Attune tibial plate. Moreover, we assessed related factors with the development of tibial RLL after the TKA. We hypothesized that the new Attune S + would have a lower incidence of RLL compared to the Attune S.

## Materials and methods

### Subjects

**Fig. 1:**
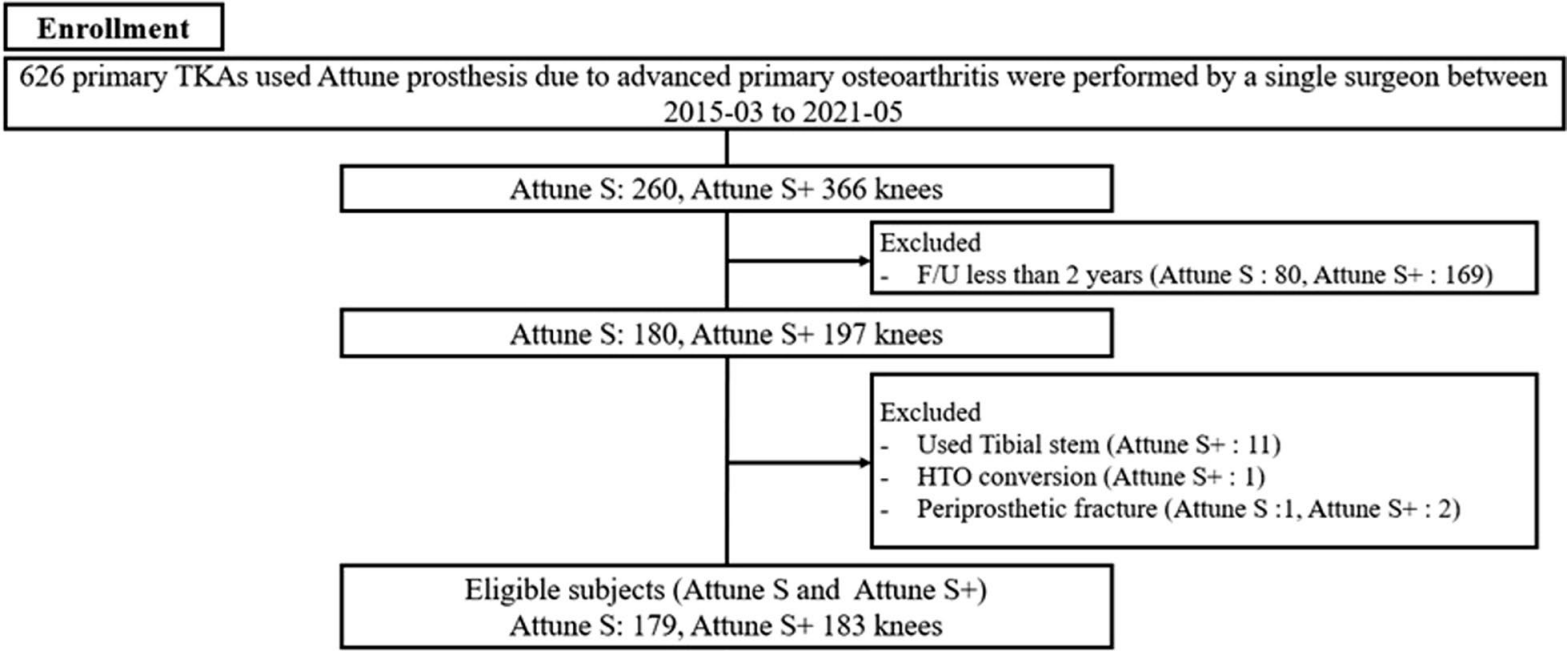
Flowchart of patient recruitment. *TKA* total knee arthroplasty, *HTO* high tibial osteotomy

The current retrospective study included 694 knees that received TKA using the ATTUNE PS prostheses from March 2015 to December 2020. The inclusion criteria were primary TKA due to advanced primary osteoarthritis with varus malalignment. The exclusion criteria were knees with (1) stemmed prostheses, (2) conversion TKA after failed high tibial osteotomy, (3) experienced periprosthetic fracture before 2 years after surgery, or (4) a follow-up period less than 2 years (Fig. 1). They were divided into the predecessor design group (Attune S, *n* = 179) and the new design group (Attune S + , n = 183). Follow-up period was inevitably longer in the Attune S group (56.7 months ± 21.3 months) than in the Attune S + group (37 months ± 11.4 months) because Attune S + has been newly introduced in 2018 in our country. In addition, to assess the related factors with the development of tibial RLL, the groups with and without RLL were divided and compared the radiologic parameters as a subgroup analysis. Present study was approved by Institutional Review Board (BLINDED). Patient consent was waived due to retrospective nature of the study design.

### Surgical techniques

All TKAs were performed by a single senior surgeon (K.-I.K.). The mid vastus approach with tourniquet pressure with 220 mmHg was used. Femoral and tibial bone were resected with modified gap balancing technique [8]. Usually, the thickness of tibial cut at the lateral plateau was aimed to 9 mm. Bone resection was performed using intramedullary guide at distal femur and extramedullary guide at proximal tibia. If there was sclerotic surface at medial side after tibial bone resection, 2-mm diameter drill holes were made to enhance cement penetration on the sclerotic cutting surface [9]. Following bone preparation and balancing, pulsed lavage was performed to remove as much debris as possible prior to cementing the components. Just before applying the bone cement, all liquid and fine debris on the surface were thoroughly removed using suction and dry gauze. Cement was applied to both surface of bone cut and under surface of the tibial implant using a cement gun with pressurization. All components were implanted using high viscosity bone cement (SmartSet, DePuy Synthes, Warsaw, IN). At closure, a suction drain was inserted and maintained for 24 h in all patients. Isometric exercises were started shortly after return to ward. Initiation of assisted range of motion (ROM) exercises, and weight-bearing ambulation with crutches were initiated the day after the surgery.

### Evaluation

**Fig. 2:**
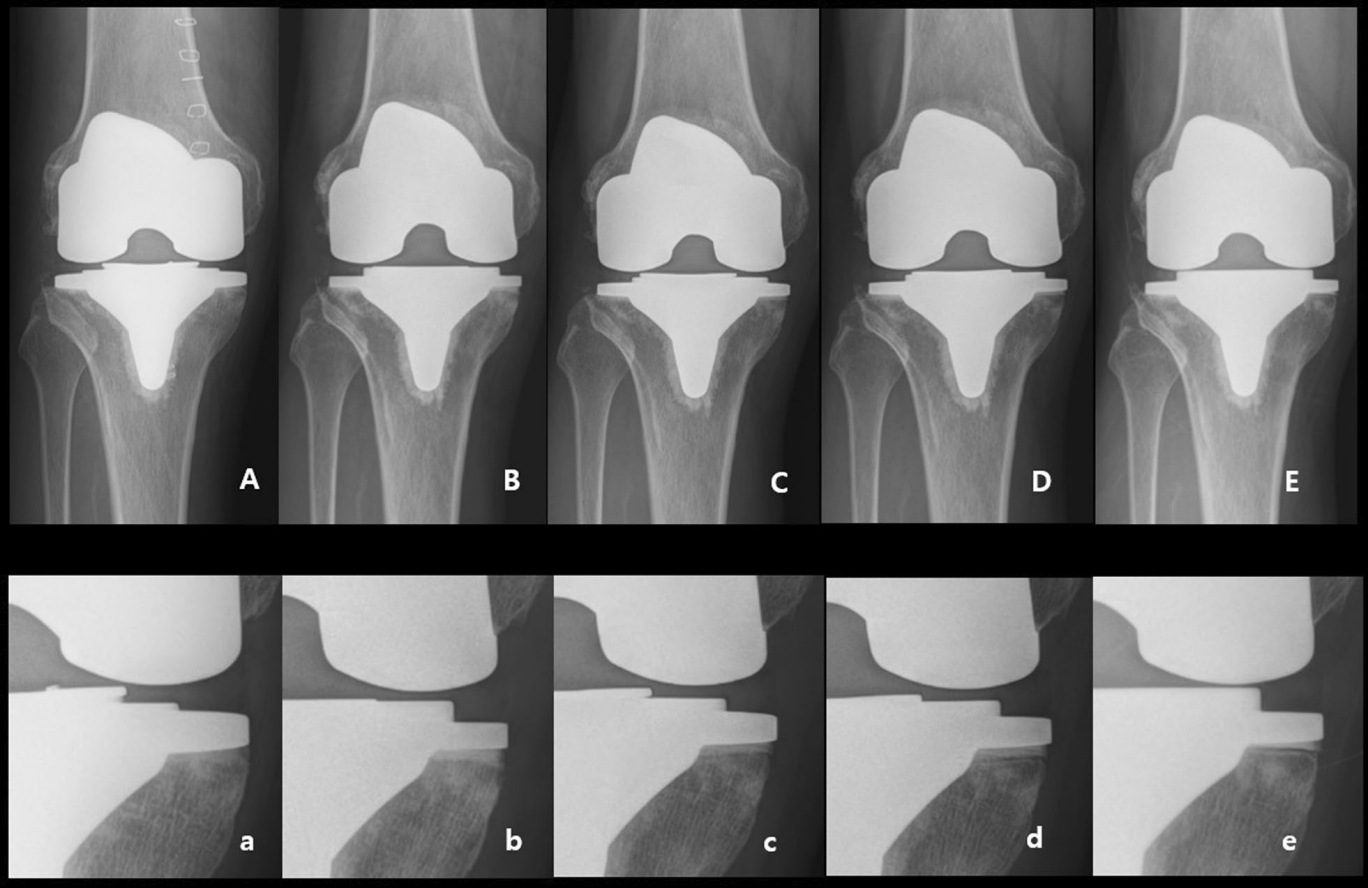
Serial anteroposterior radiographs of a 70-year-old female patient who underwent surgery using the Attune S +. Radiolucent line in cement–bone interface was visible at medial proximal tibia starting from the 6 months follow up after surgery. **A**, immediate postoperative; **B**, 6 weeks after surgery; **C**, 6 months after surgery; **D**, 1 year after surgery; **E**, 2 years after surgery

**Fig. 3:**
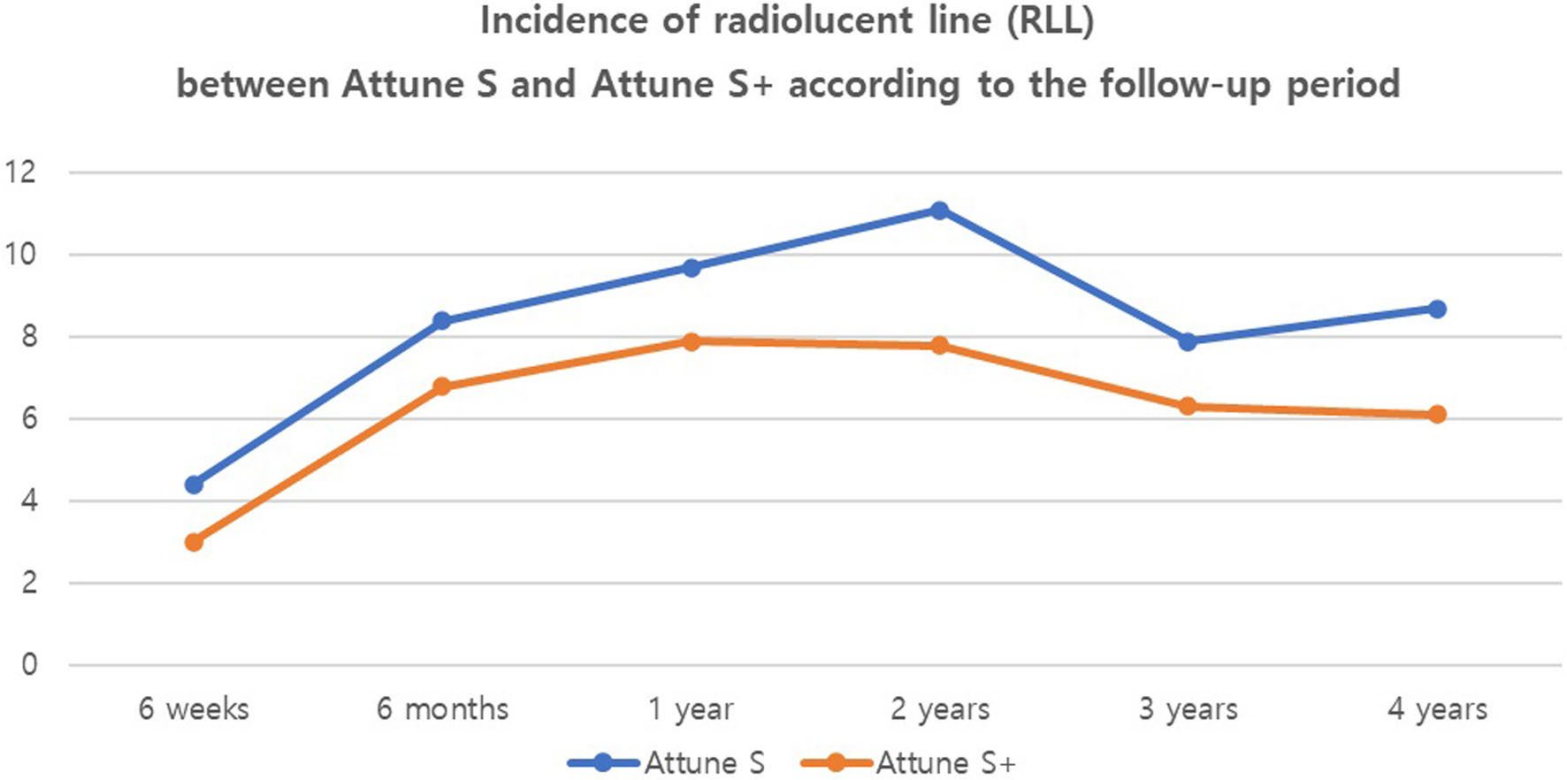
Trends of the incidence of radiolucent line between Attune S and Attune S + over time

The demographics including age, sex, body mass index (BMI), and follow-p period were compared between two groups. For clinical evaluation, ROM and the Western Ontario and McMaster Universities Osteoarthritis Index (WOMAC) [10] score were evaluated before and 2 years after surgery. Any complications were investigated until the last follow-up visit based on the standardized list, and the definitions of complications associated with TKA suggested by the Knee Society [11]. Anteroposterior (AP), lateral, full-length standing radiographs were taken at preoperative and postoperative 6 weeks, 6 months, 12 months, and then every follow-up year. A standardized protocol for radiographic evaluation was applied for all patients as the current study was performed in a single institute. Preoperative medial proximal tibial angle (MPTA), preoperative and postoperative hip-knee-ankle angle (HKAA) and the position of both components were evaluated using the Knee Society radiological evaluation method [12]. The RLL was defined as radiolucent intervals between the cement and either the implant or the bone [13]. It was regarded as being present when the bone adjacent to the radiolucency was sclerotic and parallel to the implant (Fig. 2) [13]. Since the radiographs should be taken horizontally with the tibial plate as much as possible, radiographs with a distance greater than 1.5 mm from the lowest part of the tibial plate to the line connecting the lower ends of the tibial plate were excluded as inadequately captured (Fig. 3) [14]. According to the Knee Society radiological evaluation method, the lower part of the tibial plate was divided into ten zones and the location of RLL was evaluated [12]. The incidence of RLL in ten zones below the tibial plate on standing AP and lateral radiographs taken 6 weeks, 6 months, 12 months, and then every follow up year after the surgery was compared between the attune S and attune S + groups. Medial proximal tibial angle (MPTA), preoperative and postoperative HKAA and postoperative position of components with α, β, γ, and Φ angles were compared at 6 weeks postoperatively. Measurements from these images were performed using a picture archiving and communication system (INFINITT, Seoul, Korea). To minimize observation bias, two independent investigators (M.K.U. and K.J.H.) performed the radiographic evaluations.

To assess the related factors with the development of tibial RLL, the cohort was divided into two groups as a subgroup analysis: those with RLL and those without RLL during the entire follow-up period, regardless of the type of tibial implant used. The MPTA, preoperative and postoperative 6 weeks HKAA, and position of components (α, β, γ, and Φ) were compared between the two subgroups.

### Data analyses

The incidence of RLL was compared using the Pearson chi-squared test. MPTA, preoperative and postoperative HKAA, α, β, γ, Φ angles, preoperative and postoperative ROM and WOMAC scores were compared between two groups (independent *t*-tests). Univariate and multivariate regression analysis was performed to determine the factors causing RLL after TKA. Statistical analyses were performed using SPSS version 25 (Chicago, IL, USA), and a *P* < 0.05 was considered statistically significant. The inter-observer reliabilities of measurement for radiological parameter such as position of components and HKAA was checked using the intraclass correlation coefficient and all values were greater than 0.8. Cohen’s kappa coefficient was evaluated to confirm the reliability of the incidence of the RLL and it was over 0.8 (Supplementary Table 1 and Table 2). Handling outliers or unexpected results were performed according to the decision-making tree based on the previous literature [15].

Post hoc power analyses using the significance set at an *α* of 0.05 were performed to determine whether the sample had sufficient power to detect significant differences. A power > 80% was considered sufficient, and all the significantly different variables met this criterion. Thus, it was determined that the present study was adequately powered.

## Results

### Clinical outcomes

There was no significant difference between Attune S and Attune S + groups in the demographics (Table 1). For the clinical outcomes, there were no significant differences in ROM and WOMAC scores (Table 2). Moreover, no implant failure or complications were observed in both groups until last follow-up.

**Table 1:** Patient demographics

	Attune S	Attune S +	*P* value
Number of knees	179	183	
Men/women (knee)	19/160	24/159	0.46
Right/left (knee)	90/89	91/92	0.92
Age	71.9 ± 5.8	71.0 ± 5.3	0.11
Body mass index	26.8 ± 3.2	26.1 ± 3.6	0.11
Follow up period (month)	56.7 ± 21.3	37.1 ± 11.4	< .001
Bone mineral density, *t*-score of femur neck	−1.5 ± 1.4	−1.6 ± 1.3	0.45

**Table 2:** Preoperative and postoperative clinical score

	Attune S (*N* = 179)	Attune S + (*N* = 183)	*P* value
Range of motion			
Preoperative extension	10.4 ± 11.8	10.5 ± 11.9	0.98
Preoperative flexion	119.0 ± 16.2	119.6 ± 14.4	0.74
Postoperative extension	0.4 ± 1.6	0.5 ± 1.8	0.57
Postoperative Ffexion	136.9 ± 7.0	136.6 ± 10.1	0.75
WOMAC score			
Preoperative	56.7 ± 11.6	54.8 ± 12.4	0.25
Postoperative	10.3 ± 14.3	7.8 ± 12.4	0.26

*WOMAC* Western Ontario and McMaster Universities Osteoarthritis Index

### Radiological outcomes

Regarding the radiologic outcomes, no significant differences were found between two groups in HKAA, MPTA, and postoperative position of components (α, β, γ, and Φ angles) (Table 3). The incidence of tibial RLL during 4 year serial follow-up was not significantly different between the groups reporting 10.0% (*n* = 18) and 8.2% (*n* = 15) in the Attune S and Attune S + groups, respectively (*P* = 0.54). Furthermore, all RLL were seen only in the AP zone 1 and were observed in the bone-cement interface. No case of RLL in the implant–cement interface was observed. Based on serial evaluations of radiographs, the incidence of RLL in AP zone 1 was not significantly different between the two groups at postoperative 6 weeks, 6 months, 1 year, 2 years, 3 years, and 4 years (*P* > 0.05) (Table 4) (Fig. 3).

**Table 3:** Radiologic evaluation between Attune S and Attune S +

	Attune S (*N* = 179)	Attune S + (*N* = 183)	*P* value
Preoperative MPTA (°)	85.1 ± 2.2	84.6 ± 2.9	0.61
Implant alignment (°)			
α	94.6 ± 2.1	94.5 ± 2.4	0.84
β	90.0 ± 1.6	90.0 ± 1.6	0.96
γ	0.6 ± 3.4	1.0 ± 2.4	0.20
Φ	87.0 ± 1.4	87.1 ± 3.0	0.80
Hip–knee–ankle angle(°)			
Preoperative	−171.7 ± 5.5	−171.4 ± 6.0	0.70
Postoperative 6 weeks	−179.0 ± 2.3	−178.9 ± 2.2	0.14

*MPTA* medial proximal tibial angle

**Table 4:** Incidence of radiolucent line (RLL) between attune S and attune S + according to the follow-up period

	Attune S	Attune S +	*P* value
Postoperative period			
6 weeks	4.4% (7/159)^a^	3.0% (5/164)	0.52
6 months	8.4 (13/154)	6.8 (11/162)	0.58
1 year	9.7 (15/155)	7.9 (13/165)	0.57
2 years	11.1 (17/153)	7.8 (14/180)	0.30
3 years	7.9 (11/139)	6.3 (6/95)	0.64
4 years	8.7 (11/126)	6.1 (4/66)	0.51
Overall^b^	10.0 (18/179)	8.2 (15/183)	0.54

^a^Number of knees which RLL occurred/number of knees followed at the follow-up period. In each follow-up period, there are cases where the outpatient was not visited or the X-ray was not taken properly, so there is a change in total number of knees

^b^Overall means that RLL occurred at least once during the follow-up period

*RLL* radiolucent line

*AP* anteroposterior

### Subgroup analysis

Meanwhile, based on the subgroup analysis comparing the knees with and without RLL, the preoperative HKAA of the limb alignment was significantly smaller in the group with RLL (−169.1° ± 3.6°) than those without RLL (171.8° ± 5.9°) (*P* = 0.01) (Table 5). Moreover, preoperative MPTA was significantly smaller (more tibia vara) in the group with RLL (83.7° ± 2.0°) than the group without RLL (85.0° ± 2.7°) (*P* = 0.01). However, there was no significant difference in ROM, WOMAC score and the position of the tibial implant (α, β, γ, and Φ) between the two groups (*P* > 0.05) (Table 5). Multivariate regression analysis demonstrated that preoperative MPTA (B, −0.199; *P* = 0.035) and HKAA (B, −0.107; *P* = 0.030) were significant factors for the occurrence of RLL following TKA (Table 6).

**Table 5:** Comparison between groups with and without radiolucent line (RLL)

	Without RLL (*N* = 329)	With RLL (*N* = 33)	*P* value
Hip–knee–ankle angle(°)			
Preoperative	−171.8 ± 5.9	−169.1 ± 3.6	0.01
Postoperative	−179.0 ± 2.3	−179.1 ± 2.1	0.76
Preoperative MPTA (°)	85.0 ± 2.7	83.7 ± 2.0	0.01
Implant alignment (°)			
α	94.5 ± 2.3	94.8 ± 1.8	0.56
β	90.0 ± 1.6	90.0 ± 1.2	0.98
γ	0.8 ± 2.9	0 ± 3.3	0.12
Φ	87.0 ± 2.4	86.6 ± 1.6	0.34
WOMAC score			
Preoperative	53.6 ± 11.1	51.7 ± 11.7	0.55
Postoperative	9.1 ± 13.5	3.1 ± 4.7	0.22
Range of motion			
Preoperative extension	9.0 ± 15.1	9.4 ± 12.5	0.99
Preoperative flexion	118.8 ± 16.3	118.8 ± 16.3	0.85
Postoperative extension	0.4 ± 1.6	0.9 ± 1.9	0.67
Postoperative flexion	136.7 ± 8.5	137.4 ± 5.2	0.18

In this table, “with RLL” group refers to cases where RLL occurred at least once during the follow-up period

*RLL* radiolucent line, *MPTA* medial proximal tibial angle, *WOMAC* Western Ontario and McMaster Universities Osteoarthritis Index

**Table 6:** Multivariate logistic regression analysis to determine factors for the occurrence of RLL

Variables	Univariate analysis		Multivariate analysis	*P* value
	B	*P* value	B	*P* value
Age	0.010	0.753		
BMI	−0.060	0.279		
Preoperative MPTA	−0.331	** < 0.001**	−0.199	**0.035**
Preoperative HKAA	−0.173	** < 0.001**	−0.107	**0.030**
α	0.049	0.557		
β	−0.003	0.981		
γ	−0.125	**0.044**	−0.101	0.123
δ	−0.057	0.347		
Postoperative HKAA	0.025	0.756		

*BMI* body mass index, *MPTA* medial proximal tibial angle, *HKAA* hip–knee–ankle angle

Bold indicates statistical significance

Multivariate regression analysis demonstrated that preoperative MPTA (B, −0.199; *P* = 0.035) and HKAA (B, −0.107; *P* = 0.030) were significant factors for the occurrence of RLL following TKA (Table 6).

## Discussion

Contrary to our initial hypothesis, the main finding of the present study was that there was no significant difference in the clinical and radiologic outcomes including the incidence of tibial RLL on serial follow-up between the new design cobalt-chrome tibial plate and predecessor. Rather than the design of tibial plate, the incidence of RLL seems significantly associated with tibial shape (MPTA) and preoperative varus alignment.

It is widely cited that radiolucent lines (RLLs) greater than 2 mm and increasing in size at the bone–cement interface are indicative of loosening [9, 16]. In contrast, while definitive criteria for RLLs at the implant–cement interface in total knee arthroplasty (TKA) have not been clearly established, early debonding might be suggested and thus warrant close attention [17]. Attune S + was developed to address debonding between the tibial component and cement mantle, which was observed in the previous design (Attune S) and led to aseptic loosening [6]. Additionally, a previous study reported a higher incidence of RLL below the tibial component in Attune S compared to the its previous design, PFC Sigma (DePuy Synthes, Warsaw, IN) [2]. To enhance implant–cement interface bonding of Attune S, the new design features an enhanced rear surface roughness and additional cement pockets [6]. After the new design implant has been introduced, there were some studies that compared previous and new design. One study demonstrated that the medial proximal tibial bone resorption was significantly decreased in Attune S + compared with Attune S at 2 years follow-up [5]. Meanwhile, unlike our study, the study evaluated medial tibial bone resorption instead of RLL [5]. The authors attributed their findings to the reduced stress shielding caused by the addition of a cement pocket on the tibial component, which resulted in a thinner component in some areas [5]. However, previous studies investigated the different tibial designs and their association with bone resorption; however, the association is still controversial [18–21] and the thickness of the nonpocketed area remains the same [6]. On the other hand, a previous cadaveric study confirmed the effectiveness of these modifications, demonstrating a higher bond strength at the implant–cement interface in the new design compared with the previous one [22]. However, no significant differences were observed in the depth of cement penetration into the bone or the quality of the cement mantle [22]. Meanwhile, previous studies have established a significant association between the cement–bone interface and the incidence of RLL [23, 24]. In these studies, this association can be attributed to poor cement mantle quality on the bone or insufficient depth of cement penetration [23, 24]. Considering that the aforementioned cadaveric study found no difference in the depth of cement penetration or quality of the cement mantle between Attune S and S + , we can assume that there would be no difference in RLL occurrence at the cement–bone interface. This is consistent with our finding of no difference in tibial RLL occurrence between the two designs.

Based on the subgroup analysis to assess the related factors of RLL, the group with RLL had significantly smaller preoperative MPTA (more tibia vara) and larger varus angle of preoperative lower limb alignment than those without RLL. These results were consistent with previous studies that patients with larger varus angle of lower limb alignment before surgery are more likely to have increased RLL, especially AP 1 zone, or aseptic loosening after TKA [25, 26]. In those patients, the medial bone might be cut thinner or even none than usual if the lateral cut is planned 9–10-mm thickness, therefore it makes more difficult to expose the trabecular bone and more likely to encounter a sclerotic surface of medial proximal tibia. The sclerotic medial proximal tibia interferes with cement penetration and interdigitation of the cement [27]. A previous study also demonstrated that there was a significant association between tibial RLL and preoperative sclerosis of medial tibial bony surface [13]. As cement penetration plays an important role in the fixation of tibial implants after TKA, insufficient cement penetration to bony surface would be a major cause of loosening [27, 28]. To overcome this, we have applied thorough bony preparation with pulsatile lavage and meticulous cementing techniques such as multiple drilling on sclerotic bone, pressurization of the cement when applying to the bone, dry-cleaning tibia cut surface using suction or pulsed lavage, and cement application to both bony surface and implant, which has currently been introduced to ensure sufficient cement penetration and reduce the incidence of RLL and implant failure [23, 28, 29]. Although we have applied those improved cementing technique, TKA with severe tibia vara or severely varus knee showed higher incidence of tibial RLL compared with less severe cases. Nevertheless, using the technique, we could not find any tibial loosening cases regardless of development of RLL at zone 1. Therefore, thorough bony preparation and meticulous cementing technique would be important when patients with preoperative lager varus angle and smaller MPTA to avoid inadequate cement fixation resulting in RLL and potential implant failure.

The present study has several limitations. Firstly, it is a retrospective study, and although we confirmed that there was no significant difference in the patients’ age, gender, and BMI, bias may have been unaccounted for, and other biases may not have been fully excluded. Secondly, the study has a relatively small sample size and as Attune S + was only recently introduced, the group has a shorter follow-up period. Additionally, this was a single-center study design. To confirm this interesting finding about implant durability, larger-sample, multicenter prospective studies would be needed. Thirdly, although we tried to take radiological images under the same conditions as much as possible and made standards to evaluate the images, even slight differences in imaging, such as different rotation or tilting of the knees, could have affected the accuracy of the evaluation. Fourthly, due to loss to follow-up, serial radiologic evaluation cannot be achieved from all patients, which might be a confounding factor in the current study. Lastly, this study was conducted in an Asian cohort of patients with a lower body mass and women predominance when compared with those in Western cohorts with knee osteoarthritis, which may limit its generalizability.

## Conclusion

The clinical and radiologic outcomes including the incidence of tibial RLL between a new design cobalt-chrome tibial plate or predecessor were not significantly different at an average of 4 years follow-up (10.0% versus 8.2%, *P* = 0.54). Meanwhile, the development of tibial RLL regardless of the design was associated with preoperative varus deformity of tibia and varus malalignment of lower limb. Our study emphasizes the clinical significance of preoperative varus deformity as a risk factor for RLL, suggesting that addressing this factor may be more critical than implant design choices.

## Supplementary Information

Supplementary file 1

## Data Availability

Data and materials are available when requested to corresponding author.

## Abbreviations

TKA: Total knee arthroplasty
PS: Posterior stabilized
RLL: Radiolucent line
MPTA: Medial proximal tibial angle
HKAA: Hip–knee–ankle angle
ROM: Range of motion
WOMAC: Western Ontario and McMaster Universities Osteoarthritis Index
AP: Anteroposterior
