# Supplementary material for: Does the clinical and radiologic outcomes following total knee arthroplasty using a new design cobalt-chrome tibial plate or predecessor different?
Source: Knee Surg Relat Res. 2024 Nov 12;36:34. doi: 10.1186/s43019-024-00239-0 (PMC11556021; doi:10.1186/s43019-024-00239-0)
Supplement: Supplementary file 1 [file 43019_2024_239_MOESM1_ESM.docx]

**Supplementary table 1. Reliability test for Cohen’s kappa coefficient of RLL evaluation on serial radiographs**

|  | 6 weeks | 6 months | 1 years | 2 years | 3 years | 4years |
| --- | --- | --- | --- | --- | --- | --- |
| k | 0.83 | 0.87 | 0.85 | 0.87 | 0.83 | 0.87 |

k, Cohen’s kappa statistic for interobserver agreement

**Supplementary table 2. Reliability test for Cohen’s kappa coefficient of radiologic evaluation**

|  | **k** |
| --- | --- |
| **Preoperative MPTA** | **0.76** |
| **Implant alignment** |  |
| **α** | **0.85** |
| **β** | **0.82** |
| **γ** | **0.72** |
| **Φ** | **0.8** |
| **Hip-knee-ankle angle** |  |
| **Preoperative** | **0.77** |
| **Postoperative 6 weeks** | **0.82** |

**k, Cohen’s kappa statistic for interobserver agreement**
